# IRAK1: oncotarget in MDS and AML

**DOI:** 10.18632/oncotarget.1880

**Published:** 2014-03-31

**Authors:** Levi J. Beverly, Daniel T. Starczynowski

**Affiliations:** Division of Hematology and Oncology, Department of Medicine, James Graham Brown Cancer Center, University of Louisville, Louisville, KY, USA; Department of Cancer and Cell Biology, University of Cincinnati; Division of Experimental Hematology and Cancer Biology, Cincinnati Children's Hospital Medical Center, Cincinnati, OH, USA

Myelodysplastic syndromes (MDS) are a collection of hematopoietic stem cell (HSC) disorders that consist of blood cytopenias, marrow dysplasia, and a predisposition to acute myeloid leukemia (AML). Approximately 30% of MDS patients go on to develop aggressive AML. MDS is fatal in a majority of patients as a result of marrow failure, immune dysfunction, and/or transformation to overt leukemia. Allogeneic HSC transplantation can be curative in MDS, but this option is suitable only in the small proportion of younger patients. Alterative treatment options for MDS include demethylating agents and immunomodulatory therapies. Disappointingly, the efficacy and durability of the remaining treatment options is variable. Targeted therapies have been effective in multiple myeloid diseases, and may also be effective in MDS by inhibiting the propagating clones.

We recently identified IRAK1 as a therapeutic target for MDS and certain subsets of AML [1]. IRAK1 mRNA is overexpressed in ~20-30% of MDS patients. More importantly, IRAK1 protein is dramatically overexpressed and found within a hyperactivated state in a majority of MDS marrow sample examined. IRAK1 is a serine/threonine kinase that mediates signals from Toll-like receptor (TLR) and Interleukin-1 Receptor (IL1R) (Figure 1). Following receptor activation, IRAK1 becomes phosphorylated which then leads to recruitment of TRAF6. This interaction between IRAK1 and TRAF6 activates NF-κB, MAPK, AKT and other pathways. The molecular source of IRAK1 overexpression and/ or hyperactivation in MDS (or AML) is not conclusive (Figure 1) [2]. Overexpression of TLR or necessary cofactors in MDS clones may result in chronic IRAK1 activation even in the absence of infection [3, 4]. Small molecule inhibitors targeting IRAK1 (IRAK1/4 Inhibitor. Amgen Inc.) have been originally developed for autoimmune and inflammatory diseases. Given that IRAK1 is hyperactivated (i.e., phosphorylated) in MDS but not normal marrow cells, we reasoned that inhibiting this complex with a small molecule inhibitor would selectively suppress the MDS-propagating clones.

**Figure:**
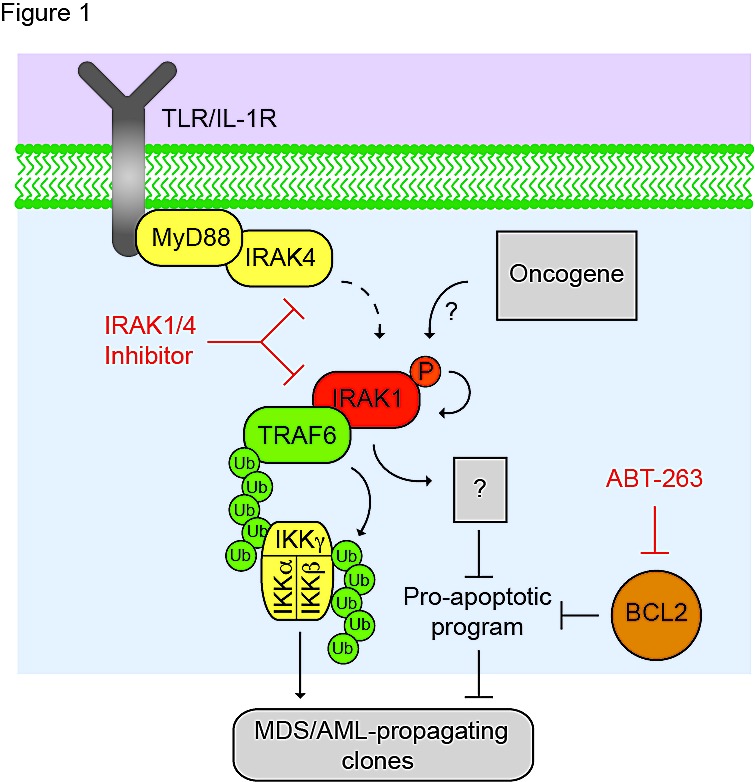
Role of IRAK1 complex in sustaining MDS- and AML-propagating cells IRAK1 is a kinase downstream from Toll-like receptor (TLR) and Interleukin-1 Receptor (IL1R). Following receptor activation under normal conditions, IRAK1 becomes phosphorylated which then leads to recruitment of TRAF6. In the context of MDS or AML, IRAK1 is activated (phosphorylated) in the absence of TLR activation, either through cross-phosphorylation or by an oncogenic kinase. Activated TRAF6 becomes autoubiquitinated by forming lysine (K)-63 linkages, which serve as a scaffold for the NF-κB kinase complex (IKKα/β/γ). TRAF6 subsequently ubiquitinates the NF-κB complex resulting in NF-κB activation. IRAK1, and likely TRAF6, have the potential to regulate other pathways relevant to sustaining the MDS/AML clones. IRAK1/4-Inh selectively targets IRAK1 and IRAK4, and inhibits TRAF6-mediated NF-κB activation. IRAK-Inh is not efficient at inhibiting anti-apoptotic family members (e.g., BCL2). To counteract this phenomenon, co-treatment with ABT-263 synergistically targets the MDS/AML-propagating cells.

We evaluated MDS and AML cell lines, as well as primary human MDS samples for sensitivity to the IRAK-Inh. MDS-propagating cells, and to a lesser extent AML cells, treated with IRAK-Inh exhibited a reduction in proliferation, progenitor function, and cell viability. To validate the effects and specificity of the IRAK-Inh, we knocked down IRAK1. As observed with the IRAK-Inh, knockdown of IRAK1 resulted in even more dramatic impairment of MDS cell proliferation, progenitor function, and viability *in vitro* and *in vivo*. To gain insight into the molecular consequences of inhibiting IRAK1 in MDS we performed gene expression profiling in MDS-derived cells. According to this analysis, IRAK1 regulates genes involved in survival, cell proliferation, RNA metabolism, cell migration/adhesion, and inflammation. Collectively, these findings implicate IRAK1 signaling in sustaining the MDS clone.

In contrast to MDS cells, AML-derived cell lines and patient samples were less sensitive to IRAK1 inhibition. This is not surprising given that AML initiating cells and blasts acquire pro-survival pathways, and therefore must overcome a greater apoptotic threshold [5]. Despite the overlap in gene signatures, we examined individual genes that would explain the discrepancy between pharmacologic and genetic approaches of IRAK1 inhibition in inducing apoptosis of MDS and AML cells. Although IRAK-Inh upregulated pro-apoptotic genes we did not observe significant alterations in the expression of anti-apoptotic BCL2-family genes. This observation prompted us to speculate that a subset of MDS/AML progenitors escape IRAK-Inh-induced apoptosis because of persistent anti-apoptotic activity of BCL2- like proteins. To test this hypothesis, we examined the survival dependence of IRAK-Inh-treated cells on BCL2 function by utilizing a BH3-mimetic (ABT-263, Abbott Laboratories). Co-treatment of the MDS or AML cells with IRAK-Inh and ABT-263 synergistically inhibited cell proliferation, progenitor function, and survival. Conversely, there was no effect on normal HSC survival or progenitor function following co-treatment with IRAK-Inh and ABT-263.

The finding that IRAK-Inh is not effective at eliminating AML clones is not surprising because in general single agent therapies have not been effective in improving survival outcomes of AML patients. In addition, the use of the BCL2 inhibitor, ABT-263, in patients has been hindered by the development of thrombocytopenia, which is an on-target effect caused by the inhibition of BCLxL. Multiple schemes can be imagined that would help combat resistance and reduce doses of the individual drugs, which may in turn diminish toxicities. The first scheme would entail developing or testing more specific drugs. To this end, new BH3- mimetic drugs (such as ABT-199) inhibit BCL2, but not BCLxL. Whether or not this new class of drugs will also be as effective as ABT-263 is still under investigation. The alternative scheme is to combine modulators of apoptosis, such as ABT-263, with known oncotargeted therapeutics [2, 6]. By pushing AML cells towards cell death by modulating anti-apoptotic BCL2 proteins, we can more effectively eliminate the AML clones while decreasing the required dose of both ABT-263 and oncotargeted agents (i.e., IRAK-Inh). Although this is not a new idea in general terms, we now specifically propose such a treatment for the elimination of MDS-propagating clones.
